# Immediate and long-term effects of zero-drop running shoes on lower extremity biomechanics

**DOI:** 10.3389/fbioe.2025.1462159

**Published:** 2025-01-22

**Authors:** Zimeng Liu, Yulin Zhou, Hui Liu, Peng Cheng, Zhiyi Zheng, Quanshou Zeng

**Affiliations:** ^1^ School of Sport Science, Beijing Sport University, Beijing, China; ^2^ China Institute of Sport and Health Science, Beijing Sport University, Beijing, China; ^3^ Key Laboratory for Performance Training and Recovery of General Administration of Sport, Beijing Sport University, Beijing, China; ^4^ Anta Sports Science Laboratory, Anta (China) Co., Ltd., Xiamen, China

**Keywords:** zero-drop running shoes, running biomechanics, strike pattern, joint work, running injury

## Abstract

**Objective:**

The purpose of the study was to investigate the immediate and long-term effects of zero-drop running shoes on lower extremity joint biomechanics.

**Methods:**

Seven male runners participated in this study (height: 1.74 ± 0.03 m, weight: 62.5 ± 3.1 kg, body mass index: 20.6 ± 0.7 kg/m^2^). Kinematic and kinetic data were collected when the participants ran at a speed of 13 ± 0.65 km/h in running shoes with zero and 15 mm drop both immediately and after the 8-week intervention wearing zero-drop running shoes. Paired t-tests were used to analyze the biomechanical differences between the different drop shoes in the immediate test and the biomechanical changes during the intervention.

**Results:**

The foot strike index increased (zero-drop: p = 0.021, 15 mm drop: p = 0.049), along with the negative work of ankle joint (15 mm drop: p = 0.018), and the hip joint (zero-drop: p = 0.004, 15 mm drop: p = 0.009), while metatarsophalangeal joint negative work decreased (zero-drop: p = 0.029, 15 mm drop: p = 0.028) in post-intervention test compared to the pre-intervention test.

**Conclusion:**

Zero-drop running shoes promote a forefoot strike pattern, which affects the distribution of lower extremity joint work.

## 1 Introduction

Running is a popular sport widely practiced by sports enthusiasts and professional athletes. In addition to focusing on performance improvement, it is also important to consider the risk of running injuries, which can affect the sports career and health of runners (Desai et al., 2021; Hollander et al., 2018; Scheer et al., 2022). Studies have shown that 70%–80% of running injuries are overuse injuries rather than immediate injuries (Walther et al., 2005). The knee, ankle, and foot are the most common sites of injury in running (Gomes Neto et al., 2023).

The biomechanics of the lower extremity during running are related to both runners’ performance and injury risk (Azeem et al., 2021; Zhang et al., 2021; Quan et al., 2023; Anderson et al., 2022; Zainuddin et al., 2022). For instance, the transition from hindfoot landing to forefoot landing may be accompanied by reductions in step length, step frequency, and other performance indicators (Azeem et al., 2021). Increased negative work of the knee joint may increase the potential risk of muscle injury around the knee joint (Hashizume et al., 2017). Furthermore, higher knee extension moments have been shown to increase peak patellofemoral joint (PFJ) stress (Zhang et al., 2022; Gu et al., 2024), which may lead to patellofemoral pain syndrome (Gu et al., 2024; Zhang et al., 2022). As a medium between the foot and the ground, the condition of running shoes could significantly impact the biomechanics of the lower extremity (Fu et al., 2022).

The heel-to-toe drop (HTD) of running shoes has significant effects on runners’ lower extremity biomechanics (Yu et al., 2022; Zhang et al., 2022; Gu et al., 2024; Malisoux et al., 2016). The HTD is defined as the difference in thickness between the heel and the forefoot of the shoes (Quan et al., 2023), which is a critical parameter of running footwear. Studies have demonstrated that wearing running shoes with a reduced HTD of approximately 15 mm immediately resulted in a 19%–24% decrease in the foot landing angle (Gu et al., 2024; Yu et al., 2022), and a reduction in knee extension moments by 9%–11% (Gu et al., 2024; Zhang et al., 2022) and the peak PFJ stress by 10%–15% (Gu et al., 2024; Zhang et al., 2022). These studies on acute tests led to a conclusion that reducing HTD of running shoes may be a potential strategy for reducing the risk of knee injuries in runners (Gu et al., 2024; Zhang et al., 2022; Zhang et al., 2021).

The long-term effects of reduced HTD on lower extremity biomechanics of runners are still not clear. The initial use of uniquely designed footwear results in higher biomechanical variability, which decreases during adaptation (Stöggl et al., 2010). Literature demonstrated that wearing zero or negative drop shoes resulted in an immediate reduction in foot landing angle (Gu et al., 2024; Yu et al., 2022). These acute effects of reduced HTD may continue to alter the foot landing angle during adaptation, thereby affecting lower extremity biomechanics. Accordingly, long-term follow-up studies on the effects of 0 or negative drop running shoes are essential. Given the higher vertical impact load rate of −8 mm drop shoes (Yu et al., 2022), some researchers suggest that choosing zero-drop shoes may be more appropriate than negative-drop shoes when implementing interventions with minimal HTD shoes (Yu et al., 2022; Davis et al., 2017).

The purpose of this study was to investigate: (1) the differences in gait parameters, lower extremity joint work and joint force between wearing zero-drop running shoes and 15 mm drop running shoes in the immediate test, (2) the changes in lower extremity biomechanics when wearing zero-drop running shoes before and after an 8-week intervention with zero-drop running shoes, and (3) the changes in lower extremity biomechanics when wearing 15 mm drop running shoes before and after the 8-week intervention with zero-drop running shoes. We hypothesized: (1) in the immediate test, the foot strike index (SI) of participants wearing zero-drop running shoes is significantly higher than when wearing 15 mm drop running shoes, (2) after long-term adaptation, the SI of participants is significantly higher than before, (3) in the immediate test, the peak PFJ stress of participants wearing zero-drop running shoes are significantly lower than when wearing 15 mm drop running shoes, (4) after long-term adaptation, the peak PFJ stress of participants is significantly lower than before, (5) in the immediate test, the knee joint negative work of participants wearing zero-drop running shoes is significantly lower than when wearing 15 mm drop running shoes, and (6) after long-term adaptation, the knee joint negative work of participants is significantly lower than before.

## 2 Methods

### 2.1 Participants

Seven male runners participated in this study (1.74 ± 0.03 m in standing height, 62.5 ± 3.1 kg in body weight, and 20.6 ± 0.7 kg/m^2^ in body mass index). Participants were included if they ran at least 10 km per week, had right-sided dominance (Li et al., 2022), and achieved a maximal oxygen uptake (VO2 max) greater than 50 mL/kg/min in a pre-test. Participants who were finally included in the study all had a weekly running volume greater than 30 km. Participants were excluded if they had suffered a lower extremity injury within 6 months prior to the study, or failed the Physical Activity Readiness Questionnaire (PAR-Q) screening.

### 2.2 Protocol

**FIGURE 1:**
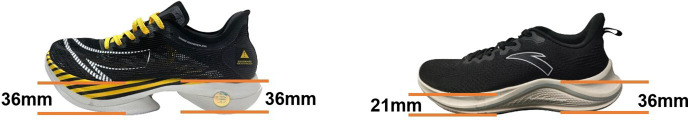
Test shoes used in the study.

Before the intervention, participants underwent an immediate test (a pre-intervention test) at a running speed of 13 ± 0.65 km/h, corresponding to approximately 70% of their velocity at VO2 max (vVO2 max). Following this, they engaged in an 8-week running intervention. During the intervention, participants were requested to complete 30 km per week using the zero-drop running shoes while maintaining their preferred training speed. To ensure they achieved the prescribed distance, a researcher monitored the participants’ daily running volume and provided reminders of the remaining weekly mileage. After completing the intervention, participants underwent a post-intervention test. The post-intervention running speed was kept the same as the pre-intervention test because research has shown that running speed significantly affects lower extremity biomechanics (Arampatzis et al., 1999). In each test, participants ran in two types of shoes with one pair of shoes having an HTD of zero, while the other had an HTD of 15 mm (Figure 1). The shoe conditions are shown in Table 1.

**TABLE 1:** Shoe conditions.

	0 mm drop running shoes	15 mm drop running shoes
Heel-to-toe Drop (mm)	0	15
Midsole Bending Stiffness (N/m)	4.3	2.0
Midsole Materials	Thermoplastic Foam (TPF)	Ethylene Vinyl Acetate (EVA)
Outsole Materials	Thermoplastic Polyurethane (CPU)	Rubber (RB)

Twenty-six reflective markers were placed bilaterally at acromion, posterior superior iliac spines, anterior superior iliac spines, lateral thighs, lateral and medial femoral condyles, anterior superior shanks, lateral and medial malleoli, toe tips, posterior calcaneus, and first and fifth metatarsophalangeal joints. An additional marker was placed between the fourth and fifth lumbar vertebrae. Each participant had a standing static calibration test. The markers at medial femoral condyles and medial malleoli were then removed.

### 2.3 Data collection

Kinematic and kinetic data were collected synchronously during the running trials using two three-dimensional force plates (Kistler 9287C, Switzerland) at a sampling frequency of 1,000 Hz and an eight-camera infrared high-speed motion capture system (Qualisys Arqus 12, Sweden) at 200 Hz. Running speed was monitored by a portable timing system (Smart Speed, Australia), with two infrared emitters placed 3 m apart on one side of the force plates.

Each participant completed three successful trials for each shoe condition in each test (Zhang et al., 2022; Yu et al., 2022). A successful trial was defined as: (1) the participant’s running speed was within the required range, (2) the entire stance phase of the dominant leg, from initial contact to toe-off, occurred on the same force plate, and (3) run across the force platform area without any obvious movement adjustment.

### 2.4 Data reduction

The three-dimensional coordinate data of the markers were filtered by a second-order Butterworth low-pass digital filter with a frequency of 13.3 Hz (Yu et al., 1999). A vertical ground reaction force of 10 N was defined as the threshold for determining the landing and toe-off (Wang et al., 2021).

Step length was defined as the distance from the initial right foot landing to the subsequent left foot landing. Step time was defined as the duration from the initial right foot landing to the subsequent left foot landing. Stance time was defined as the duration from the initial right foot landing to the initial right foot toe-off. The SI was calculated to quantify the foot strike pattern of the runners, as described by Cavanagh (Cavanagh and Lafortune, 1980). A higher SI indicates a more forefoot strike pattern. The SI was calculated as:

$$SI=\frac{D_{COP-Heel}}{Foot\;length}\times 100\%$$

where D_COP−Heel_ represented the distance between the center of pressure (COP) and the heel at the moment of initial right foot contact. Foot length was defined as the distance from the heel marker to the toe marker.

The definitions of lower extremity joint center, joint angles, and angular velocities were consistent with literature (Wu et al., 2002; Willwacher et al., 2013). The net joint moment of the hip, knee and ankle joints were calculated using inverse dynamics (Winter 2009). The net joint moment of the metatarsophalangeal (MTP) joint was calculated using the method described in Cigoja’s study (Cigoja et al., 2019). Joint power was defined as the product of the net joint moment and the angular velocity of the joint, and the trapezoidal method was used to calculate the joint power as an integral over time to obtain the joint work (Willwacher et al., 2013).

Studies have shown that frontal and transverse planes movements have minimal impact on the kinetic indicators during running (Harrison et al., 1986; Williams, 1985). Therefore, only sagittal plane data were analyzed in this study. This study calculated the net work, negative work, and positive work for the hip, knee, ankle, and MTP joints. Patellofemoral joint contact force and stress were calculated as described by Whyte and Vannatta (Whyte et al., 2010; Vannatta and Kernozek, 2015). The Achilles tendon (AT) force during running was approximated as the peak plantar flexion force, calculated as the ratio of the peak plantar flexion moment to the AT moment arm as described by Lyght and Rugg (Lyght et al., 2016; Rugg et al., 1990). All joint force and work results were normalized by their own body weight (BW).

### 2.5 Data analyses

To test hypotheses 1 to 3, paired t-tests were used to compare the differences in lower extremity biomechanics when participants wore zero-drop running shoes compared to 15 mm drop running shoes in pre-intervention test. To test hypotheses 4 to 6, paired sample t-tests were used to compare the differences in lower extremity biomechanics between pre- and post-intervention tests. It should be noted that this study did not involve multiple comparisons. Statistical testing results with Type I error rate less than 0.05 were considered as statistically significant. All data analyses were performed using SPSS Computer Program Package Version 26.0 (SPSS Science, Chicago, IL, United States). Effect sizes were calculated using Cohen’s d, with Cohen’s d < 0.5 being defined as “small”, 0.5 ≤ Cohen’s d ≤ 0.8 as “medium”, and Cohen’s d > 0.8 as “large”.

## 3 Results

In pre-intervention test, no significant differences were observed in SI (t = 1.821, p = 0.118, Cohen’s d = 0.74), step length (t = −0.354, p = 0.736, Cohen’s d = 0.14), step time (t = 1.100, p = 0.314, Cohen’s d = 0.45), and stance time (t = −0.147, p = 0.888, Cohen’s d = 0.06) for participants wearing zero-drop running shoes compared to 15 mm drop running shoes. In post-intervention test, the SI was significantly higher (zero-drop: p = 0.021, 15 mm drop: p = 0.049), and the stance time (zero-drop: p = 0.008, 15 mm drop: p = 0.005) was significantly lower compared to the pre-intervention test. No significant difference was observed in step length between pre- and post-intervention tests (zero-drop: p = 0.070, 15 mm drop: p = 0.967). In post-intervention test, the step time of zero-drop running shoes was significantly lower compared to the pre-intervention test (p = 0.032). No significant difference was observed in step time of 15 mm drop running shoes between pre- and post-intervention tests (p = 0.814) (Table 2).

**TABLE 2:** Gait indicators.

Variables	Shoes	Pre-intervention	Post-intervention	t	P-value	Cohen’s d
SI (%)	0 mm drop	54% ± 25%	69% ± 12%	−3.097	**0.021**	1.25
	15 mm drop	38% ± 30%	50% ± 26%	−2.457	**0.049**	0.98
Stance time (s)	0 mm drop	0.21 ± 0.01	0.19 ± 0.01	3.850	**0.008**	1.57
	15 mm drop	0.21 ± 0.01	0.19 ± 0.02	4.329	**0.005**	1.77
Step distance (m)	0 mm drop	1.33 ± 0.08	1.31 ± 0.09	2.201	0.070	0.90
	15 mm drop	1.34 ± 0.07	1.34 ± 0.05	−0.043	0.967	0.02
Step time (s)	0 mm drop	0.36 ± 0.02	0.34 ± 0.02	2.791	**0.032**	1.14
	15 mm drop	0.36 ± 0.02	0.35 ± 0.02	0.246	0.814	0.10

Bold P-Values indicate a significant difference (p < 0.05) between pre- and post-intervention tests.

In pre-intervention test, the peak PFJ stress (t = −3.625, p = 0.011, Cohen’s d = 1.48) was significantly lower when participants wore zero-drop running shoes compared to 15 mm drop running shoes. No significant differences were observed in AT force (t = −0.126, p = 0.904, Cohen’s d = 0.05) and PFJ force (t = −1.845, p = 0.115, Cohen’s d = 0.94) between wearing zero-drop running shoes and 15 mm drop running shoes. In post-intervention test, the peak PFJ stress (zero-drop: p = 0.011, 15 mm drop: p = 0.004) was significantly higher compared to the pre-intervention test. No significant differences were observed in PFJ force (zero-drop: p = 0.056, 15 mm drop: p = 0.086) and AT force (zero-drop: p = 0.606, 15 mm drop: p = 0.213) between pre- and post-intervention tests (Table 3).

**TABLE 3:** Joint force and stress.

Variables	Shoes	Pre-intervention	Post-intervention	t	P-value	Cohen’s d
The peak PFJ stress (BW)	0 mm drop	8.94 ± 1.57	11.94 ± 2.39	−3.598	**0.011**	1.47
	15 mm drop	10.33 ± 1.60	12.73 ± 1.42	−4.485	**0.004**	1.83
PFJ force (BW)	0 mm drop	4.08 ± 0.96	5.08 ± 1.50	−2.369	0.056	0.97
	15 mm drop	4.93 ± 1.19	5.84 ± 0.90	−2.050	0.086	0.84
AT force (BW)	0 mm drop	7.20 ± 0.36	7.59 ± 0.89	−0.543	0.606	0.22
	15 mm drop	7.24 ± 0.79	8.01 ± 0.56	−1.395	0.213	0.57

Bold P-Values indicate a significant difference (p < 0.05) between pre- and post-intervention tests.

In pre-intervention test, the net work (t = 2.988, p = 0.024, Cohen’s d = 1.22) and negative work (t = 3.554, p = 0.012, Cohen’s d = 1.45) of hip joint were significantly lower when participants wore zero-drop running shoes compared to 15 mm drop running shoes. No significant differences were observed in hip joint positive work between zero-drop and 15 mm drop running shoes (t = 0.305, p = 0.771, Cohen’s d = 0.12). In post-intervention test, the positive work (zero-drop: p = 0.014, 15 mm drop: p = 0.005) and negative work (zero-drop: p = 0.004, 15 mm drop: p = 0.009) of hip joint were significantly greater compared to the pre-intervention test. No significant differences were observed in hip joint net work between pre- and post-intervention tests (zero-drop: p = 0.735, 15 mm drop: p = 0.134) (Table 4).

**TABLE 4:** Hip joint work.

Variables	Shoes	Pre-intervention	Post-intervention	t	P-value	Cohen’s d
Net Work (J/BW)	0 mm drop	−0.001 ± 0.017	0.003 ± 0.024	−0.355	0.735	0.15
	15 mm drop	−0.009 ± 0.012	0.008 ± 0.022	−1.730	0.134	0.71
Positive Work (J/BW)	0 mm drop	0.014 ± 0.010	0.040 ± 0.017	−3.409	**0.014**	1.39
	15 mm drop	0.013 ± 0.006	0.050 ± 0.023	−4.288	**0.005**	1.75
Negative Work (J/BW)	0 mm drop	−0.015 ± 0.008	−0.037 ± 0.012	4.489	**0.004**	1.83
	15 mm drop	−0.022 ± 0.012	−0.042 ± 0.008	3.780	**0.009**	1.54

Bold P-Values indicate a significant difference (p < 0.05) between pre- and post-intervention tests.

In pre-intervention test, the knee joint negative work was significantly lower when participants wore zero-drop running shoes compared to 15 mm drop running shoes (t = 2.787, p = 0.032, Cohen’s d = 1.14). No significant differences were observed in net work (t = 1.884, p = 0.109, Cohen’s d = 0.77) and positive work (t = −3.392, p = 0.190, Cohen’s d = 0.60) of knee joint between zero-drop and 15 mm drop running shoes. In post-intervention test, the knee joint net work was significantly lower (zero-drop: p = 0.015, 15 mm drop: p = 0.040), and the knee joint positive work was significantly greater compared to the pre-intervention test (zero-drop: p = 0.002, 15 mm drop: p = 0.040). No significant differences were observed in knee joint negative work between pre- and post-intervention tests (zero-drop: p = 0.942, 15 mm drop: p = 0.767) (Table 5).

**TABLE 5:** Knee joint work.

Variables	Shoes	Pre-intervention	Post-intervention	t	P-value	Cohen’s d
Net Work (J/BW)	0 mm drop	−0.019 ± 0.010	−0.008 ± 0.007	−3.390	**0.015**	1.38
	15 mm drop	−0.023 ± 0.011	−0.012 ± 0.009	−2.615	**0.040**	1.07
Positive Work (J/BW)	0 mm drop	0.024 ± 0.007	0.035 ± 0.007	−5.324	**0.002**	2.17
	15 mm drop	0.029 ± 0.009	0.038 ± 0.004	−2.514	**0.046**	1.03
Negative Work (J/BW)	0 mm drop	−0.043 ± 0.013	−0.043 ± 0.012	0.076	0.942	0.03
	15 mm drop	−0.052 ± 0.018	−0.050 ± 0.009	−0.310	0.767	0.13

Bold P-Values indicate a significant difference (p < 0.05) between pre- and post-intervention tests.

In pre-intervention test, no significant differences were observed in net work (t = 1.057, p = 0.331, Cohen’s d = 0.43), positive work (t = 0.280, p = 0.789, Cohen’s d = 0.11), and negative work (t = 0.508, p = 0.629, Cohen’s d = 0.21) of ankle joint when participants wore zero-drop running shoes compared to 15 mm drop running shoes. In post-intervention test, the ankle joint net work was significantly lower compared to the pre-intervention test (zero-drop: p = 0.004, 15 mm drop: p = 0.002). No significant difference was observed in ankle joint positive work between pre- and post-intervention tests (zero-drop: p = 0.301, 15 mm drop: p = 0.253). No significant difference was observed in ankle joint negative work of zero-drop running shoes between pre- and post-intervention tests (p = 0.210). In post-intervention test, the ankle joint negative work of 15 mm drop running shoes was significantly lower compared to the pre-intervention test (p = 0.018) (Table 6).

**TABLE 6:** Ankle joint work.

Variables	Shoes	Pre-intervention	Post-intervention	t	P-value	Cohen’s d
Net Work (J/BW)	0 mm drop	0.014 ± 0.010	−0.001 ± 0.004	4.459	**0.004**	1.82
	15 mm drop	0.101 ± 0.015	−0.011 ± 0.008	5.075	**0.002**	2.07
Positive Work (J/BW)	0 mm drop	0.084 ± 0.015	0.077 ± 0.009	1.132	0.301	0.46
	15 mm drop	0.082 ± 0.012	0.076 ± 0.007	1.265	0.253	0.52
Negative Work (J/BW)	0 mm drop	−0.070 ± 0.019	−0.078 ± 0.010	1.405	0.210	0.57
	15 mm drop	−0.072 ± 0.018	−0.087 ± 0.013	3.220	**0.018**	1.31

Bold P-Values indicate a significant difference (p < 0.05) between pre- and post-intervention tests.

In pre-intervention test, the net work (t = 6.158, p = 0.001, Cohen’s d = 2.51), positive work (t = 0.382, p = 0.015, Cohen’s d = −1.38), and negative work (t = 6.117, p = 0.001, Cohen’s d = 2.50) of MTP joint were significantly lower when participants wore zero-drop running shoes compared to 15 mm drop running shoes. In post-intervention test, the net work (zero-drop: p = 0.041, 15 mm drop: p = 0.044), positive work (zero-drop: p = 0.008, 15 mm drop: p = 0.001), and negative work (zero-drop: p = 0.029, 15 mm drop: p = 0.029) of MTP joint were significantly lower compared to the pre-intervention test (Table 7).

**TABLE 7:** MTP joint work.

Variables	Shoes	Pre-intervention	Post-intervention	t	P-value	Cohen’s d
Net Work (J/BW)	0 mm drop	−0.057 ± 0.036	−0.033 ± 0.023	−2.593	**0.041**	1.06
	15 mm drop	−0.164 ± 0.061	−0.123 ± 0.024	−2.514	**0.045**	1.03
Positive Work (J/BW)	0 mm drop	0.004 ± 0.003	0.001 ± 0.001	3.888	**0.008**	1.59
	15 mm drop	0.009 ± 0.003	0.002 ± 0.002	5.707	**0.001**	2.33
Negative Work (J/BW)	0 mm drop	−0.062 ± 0.037	−0.034 ± 0.024	−2.848	**0.029**	1.16
	15 mm drop	−0.173 ± 0.062	−0.124 ± 0.025	−2.883	**0.028**	1.18

Bold P-Values indicate a significant difference (p < 0.05) between pre- and post-intervention tests.

## 4 Discussion

This study investigated the immediate and long-term effects of zero-drop running shoes on lower extremity joint biomechanics. The results of this study did not support our first hypothesis that in the immediate test, the SI of participants wearing zero-drop running shoes would be higher than that of those wearing 15 mm drop running shoes. The results of this study did support the second hypothesis that the SI would increase after long-term adaptation. The results of this study supported our third hypothesis that, in the immediate test, participants wearing zero-drop running shoes had significantly lower peak PFJ stress compared to those wearing 15 mm drop running shoes. However, the study did not support our fourth hypothesis that peak PFJ stress would be significantly lower after long-term adaptation. The results of this study supported our fifth hypothesis that, in the immediate test, participants wearing zero-drop running shoes had significantly less negative knee joint work compared to those wearing 15 mm drop running shoes. The results of this study did not support our sixth hypothesis that participants’ knee joint negative work would decrease further after adaptation. In conclusion, after long-term adaptation, there was a more forward foot strike pattern, redistribution of lower extremity joint work, and changes in PFJ stress, which may affect runners’ performance and injury risk.

For foot strike pattern, previous studies demonstrated that zero or negative drop running shoes could reduce the foot landing angle immediately (Zhang et al., 2022; Gu et al., 2024). However, some studies suggested that changing running shoes does not immediately change the foot strike pattern (Mullen et al., 2019). These inconsistent results were possibly due to differences in shoe design and test conditions. In addition to HTD, cushioning is also an influential factor in foot strike pattern. Specifically, the better the cushioning, the more likely a runner is to adopt a rearfoot strike pattern (Squadrone et al., 2015). In terms of sole materials, the zero-drop running shoes used in this study have a lower cushioning effect, while the 15 mm drop shoes have a higher cushioning effect. However, the acute test results in this study showed no significant differences in SI between these two shoes. After long-term adaptation, participants’ foot strike pattern shifted forward, with a 15% increase in SI for zero-drop running shoes and a 12% increase for 15 mm drop running shoes. Previous research has shown that the effect of shoe cushioning on lower extremity biomechanics is independent of adaptation time (Agresta et al., 2018). Therefore, we believe that the observed changes in foot strike patterns following the intervention were primarily driven by adaptation to the zero-drop design, which subsequently influenced lower extremity biomechanics. While forefoot strike patterns have been suggested to reduce impact and push-off force (Mullen et al., 2019), recent prospective research shows no definitive link between foot strike patterns and overall injury risk (Dillon et al., 2023). However, specific injuries, such as increased knee joint stress linked to rearfoot strike pattern and Achilles tendon force associated with forefoot strike patterns, remain important considerations (Dillon et al., 2023). Meanwhile, stance time decreased significantly after long-term adaptation, suggesting that zero-drop running shoes may alter gait patterns. Gait retraining could be widely used to treat running-related injuries (Moran and Wager, 2020).

For joint force, in the immediate test, wearing zero-drop running shoes resulted in a 13% reduction in peak PFJ stress compared to 15 mm drop running shoes, consistent with previous studies. Research demonstrated that shoes with a 10–16 mm HTD reduction could reduce the peak PFJ stress by 9%–16% (Gu et al., 2024; Zhang et al., 2022), primarily due to the reduction in knee joint extension moment (Zhang et al., 2022). These studies suggested that zero or negative HTD shoes effectively reduced the peak PFJ stress, thereby reducing injury risk. Previous studies have demonstrated that increased shoe cushioning is associated with a reduction in joint forces (Meardon et al., 2018). The results of this study suggest that less cushioned, zero-drop running shoes effectively reduced the peak PFJ stress of runners. This indicates that shoe cushioning may not be the primary factor influencing joint force, and other design features such as HTD may play a significant role. After long-term adaptation, the peak PFJ stress increased, possibly due to an increase in lower extremity strength during adaptation to zero-drop running shoes. Previous studies have demonstrated that different strength training methods affect knee extension moments (Aagaard et al., 1994), which in turn affect the peak PFJ stress (Zhang et al., 2022).

For joint work, in the immediate test, wearing zero-drop running shoes resulted in a 17% reduction in negative knee joint work compared to 15 mm drop running shoes. This suggested that zero-drop shoes could reduce the knee joint load during running, thereby decreasing knee joint injury risk and muscle fatigue (Hashizume et al., 2017). No significant change was observed in knee joint negative work between pre- and post-intervention tests, indicating that the reduction in negative work with zero-drop running shoes was unrelated to adaptation. Additionally, results also showed that after long-term adaptation, participants’ ankle joint negative work increased. Studies have demonstrated that a forefoot strike pattern would increase ankle joint negative work and decrease knee joint negative work (Hamill et al., 2014). Negative joint work was associated with a higher risk of muscle injury around the joint (Hashizume et al., 2017). Therefore, runners with poor ankle strength might not be suitable for zero-drop running shoes. In acute tests, the positive and negative work of the MTP joint was lower when wearing zero-drop running shoes. Moreover, after intervention, it was further reduced. Research has found that zero-drop running shoes could reduce MTP joint load and save energy output (Martinez et al., 2024), which is consistent with the results of our study. In this study, besides the significant difference in HTD, the zero-drop shoes had greater stiffness compared to the 15 mm drop shoes. Previous studies have indicated that shoe stiffness primarily affects MTP joint work (Liu et al., 2024). And stiffer shoes leading to a reduction in MTP negative work (Cigoja et al., 2019; Liu et al., 2024) but increase in MTP positive work (Cigoja et al., 2019), which is not entirely consistent with our result. Overall, the sustained decrease in MTP positive and negative work after long-term adaptation may be attributed to the combined effects of higher shoe stiffness and lower HTD, and is mainly influenced by HTD. Research demonstrated that changes in MTP joint power are potential mechanisms for the redistribution of lower extremity joint work (Cigoja et al., 2019). In this study, hip joint negative work, hip joint positive work, and knee joint positive work were increased after long-term adaptation, indicating greater energy output and load at hip joint and greater energy output at the knee joint. The results of this study showed that runners’ lower extremity joint work was redistributed after long-term adaptation.

This study provides a preliminary examination of the effects of long-term adaptation to zero-drop running shoes on lower extremity biomechanics. Several limitations of this study should be acknowledged. First, the running speed during the test was constant, whereas running speed significantly affects lower extremity biomechanics (Arampatzis et al., 1999). Second, we did not measure changes in muscle strength around the joints before and after the intervention, so conclusions about injury risk should be treated with caution and warrant further investigation. Previous studies have shown that, in addition to footwear, running surface type also influences lower extremity biomechanics (Yang et al., 2024). Therefore, future research should focus on examining lower extremity biomechanics under varying running speeds and surface conditions to better inform footwear selection based on running conditions. In addition, lower extremity stiffness would influence the running performance (Ziliaskoudis et al., 2019). Future research can further explore the impact of zero-drop running shoes on lower extremity stiffness.

## 5 Conclusion

This study found that long-term wearing of zero-drop running shoes resulted in a more forefoot strike pattern in runners. While wearing zero-drop running shoes could immediately reduce the peak PFJ stress, long-term wearing could increase this stress. Long-term wearing of zero-drop running shoes affected the distribution of lower extremity joint work, specifically decreasing the load on the MTP joint while increasing the load on the hip and ankle joints.

## Data Availability

The raw data supporting the conclusions of this article will be made available by the authors, without undue reservation.
